# Silencing ELMO3 Inhibits the Growth, Invasion, and Metastasis of Gastric Cancer

**DOI:** 10.1155/2018/3764032

**Published:** 2018-09-24

**Authors:** Yan Hu, Qiongfang Yu, Yao Zhong, Wei Shen, Xiaoyan Zhou, Xu Liu, Mingpu Xu, Nanjin Zhou, Weiping Min, Dian Gao

**Affiliations:** ^1^Department of Pathogen Biology and Immunology, Medical College of Nanchang University, Nanchang, Jiangxi 330006, China; ^2^Department of Gastroenterology and Hepatology, Second Affiliated Hospital of Nanchang University, Nanchang, Jiangxi 330006, China; ^3^Department of Gastrointestinal Surgery, Second Affiliated Hospital of Nanchang University, Nanchang, Jiangxi 330006, China; ^4^Department of Pathophysiology, Medical College of Nanchang University, Nanchang, Jiangxi 330006, China; ^5^Second Clinical Medical College of Nanchang University, Nanchang, Jiangxi 330006, China; ^6^Institute of Molecular Medicine, Jiangxi Academy of Medical Sciences, Nanchang, Jiangxi 330006, China

## Abstract

ELMO3 is a member of the engulfment and cell motility (ELMO) protein family, which plays a vital role in the process of chemotaxis and metastasis of tumor cells. However, remarkably little is known about the role of ELMO3 in cancer. The present study was conducted to investigate the function and role of ELMO3 in gastric cancer (GC) progression. The expression level of ELMO3 in gastric cancer tissues and cell lines was measured by means of real-time quantitative PCR (qPCR) and Western blot analysis. RNA interference was used to inhibit ELMO3 expression in gastric cancer cells. Then, wound-healing assays, Transwell assays, MTS assays, flow cytometry, and fluorescence microscopy were applied to detect cancer cell migration, cell invasion, cell proliferation, the cell cycle, and F-actin polymerization, respectively. The results revealed that ELMO3 expression in GC tumor tissues was significantly higher than in the paired adjacent tissues. Moreover, knockdown of ELMO3 by a specific siRNA significantly inhibited the processes of cell proliferation, invasion, metastasis, regulation of the cell cycle, and F-actin polymerization. Collectively, the results indicate that ELMO3 participates in the processes of cell growth, invasion, and migration, and ELMO3 is expected to be a potential diagnostic and prognostic marker for GC.

## 1. Introduction

Gastric cancer (GC) is a common global malignant tumor that occurs in the gastric mucosa [1]. It causes a serious threat to human health and its prognosis is relatively poor. According to statistics from the International Cancer Research Institute [2], metastatic spread of GC is still the primary cause of death of afflicted patients, although advances have been made in the diagnosis and treatment of GC. Therefore, it is important to explore the molecular mechanisms correlated with the recurrence or metastasis of GC.

As early as 1863, Rudolf Virchow, a German pathologist, proposed that tumor cells have amoebic motility and chemotaxis [3, 4]. Cell migration is integral to the whole process of tumor cell metastasis. Chemotaxis is a directional form of cell migration mediated by a series of chemokine gradient processes that are involved in numerous physiological processes such as recruitment of neutrophils, metastasis of tumor cells, and development of the model organism* Dictyostelium discoideum *[5]. The movement of cancer cells and leukocyte chemotactic movement are similar to the cell pseudopodia stretching process. In eukaryotes, signaling modulating cell movement is initiated by chemokine receptors, specifically, a subfamily of G protein-coupled receptors (GPCRs) [6]. GPCRs bind chemokine chemoattractants to promote the dissociation of heterotrimeric G-proteins. Then, the active G*α*i and G*βγ* subunits of G-protein in turn interact with various downstream effector molecules, which results in the activation of the small GTPase Rac. This leads to actin polymerization by promoting the growth of actin filaments and drives cell migration [5, 7]. However, how the GPCR/G*α*G*βγ* signaling network is linked to Rac activation in cell migration is not fully clear.

The engulfment and cell motility (ELMO) protein family plays a critical role in the Rac-controlled actin cytoskeleton rearrangement. ELMO is evolutionarily conserved and was first identified as CED-12 in* Caenorhabditis elegans *[8, 9]. In the eukaryotic model organism* D. discoideum, *the ELMO family was identified as having six new members, named ElmoA–F [10, 11]. The ELMO protein family in mammals consists of three transcription splicing bodies: ELMO1, ELMO2, and ELMO3. Previous studies have confirmed both distinct and identical functions of ELMO1 and ELMO2 in cell migration, cell phagocytosis, and myoblast fusion [5, 12]. They interact with the Dock family to form ELMO/Dock180 complexes, which control Rac-mediated cytoskeletal dynamics during cell migration [13–17]. Some molecules, such as Axl [18] and GPCR signaling [6], involving in regulating or interacting with ELMOs contribute to the regulatory progress.

Compared with ELMO1 and ELMO2, the biological functions of ELMO3 remain largely unknown. The expression of ELMO3 has been reported in lung cancer [19–22], colorectal cancer [23], head and neck squamous cell carcinomas [24], and laryngeal cancer [25], etc. In these cancers, upregulation of ELMO3 is commonly involved in tumor development and metastasis formation. Although ELMO3 is similar to ELMO1 and ELMO2 at the amino acid level, it is not clear whether ELMO3 possesses a similar molecular regulatory mechanism. Furthermore, in non-small-cell lung cancer, high expression of ELMO3 is found in combination with low methylation levels of its promoter region, which are associated with the formation of metastases [21]. However, in human glioblastomas, no significant association is found between the methylation level of the ELMO3 promoter and overall survival [26]. It is possible that the regulatory mechanism of ELMO3 in cancers depends on the tumor type. Currently, there are no reports about the function and molecular regulation of ELMO3 in gastric cancer.

In the present study, we hypothesized that ELMO3 may play a central role in cell proliferation, invasion, and metastasis of GC. Therefore, we compared the expression level of ELMO3 in gastric cancer tissue with that in matched adjacent normal tissues. We further investigated the potential function of ELMO3 in cell proliferation, cell cycle, invasion, and migration of SGC 7901 and BGC 823 cells. These findings may lead to a better understanding of the role of ELMO3 in GC progression.

## 2. Material and Methods

### 2.1. Patients and Samples

This study was conducted according to the Declaration of Helsinki. It was approved by the ethics committee of the Second Affiliated Hospital of Nanchang University (No. 201420). Tissue specimens of tumor tissues and adjacent normal mucosa were collected from 41 patients who underwent gastric resection for GC at the Department of Surgery, the Second Affiliated Hospital of Nanchang University from January 2015 to November 2016. The tissue samples were divided into three parts, one of which was fixed with paraformaldehyde for hematoxylin-eosin (HE) staining analysis. The remaining samples were immediately frozen in liquid nitrogen and subsequently stored at −80°C for reverse transcription-polymerase chain reaction and western blot analysis. All of the patients included in the present study had complete data, without records of having tumors in any other areas, and had not received any chemotherapy or radiation therapy previously.

### 2.2. Real-Time Reverse Transcription-Polymerase Chain Reaction

The mRNA transcript level of ELMO3 was detected in both gastric cancer tissue samples and cell lines, including gastric cancer cell lines SGC7901, BGC823, MGC803, AGS, and MKN74 and one normal gastric epithelial cell line, Ges-1. Total RNA was extracted from tissues/cells with TRIzol reagent (Invitrogen, Life Technologies, Carlsbad, CA, USA) according to the manufacturer's instructions. Then, 2 *µ*g of total RNA as a template was used to generate cDNA with a cDNA synthesis kit (Promega Corporation, Madison, WI, USA). The real-time reverse transcription-polymerase chain (qPCR) reaction was carried out with specific primers as per the protocol described in our previous report [23].

### 2.3. Western Blot Analysis

To determine the protein level of ELMO3, Western blot analysis was conducted as previously described [23, 27]. Briefly, 35 *μ*g of total protein was resolved by 12% SDS-PAGE gel electrophoresis followed by transfer onto PVDF membranes. Then, the membranes were probed with primary antibodies against ELMO3 (1:150; Thermo Fisher Scientific, Rockford, IL, USA) overnight at 4°C. After that, the membranes were further incubated for 2 h with goat anti-mouse IgG conjugated with peroxidase (Beyotime, Beijing, China). Finally, the immunoblot was developed with ECL detection reagents (Thermo Fisher Scientific, Rockford, IL, USA). The same membranes were stripped and reprobed with a primary antibody against *β*-actin, which was used as an internal control. The protein level was normalized to *β*-actin.

### 2.4. Cell Culture and Transfection

Gastric cells were cultured in RPMI Medium 1640 supplemented with 10% FBS. All cells were incubated at 37°C with 5% CO_2_. The ELMO3 siRNA and negative control were designed and synthesized by the GenePharma Company (Shanghai, China). Then, the SGC7901 and BGC 823 cells were transfected with these siRNAs and negative controls with Lipofectamine TM 2000 reagent (Invitrogen, Carlsbad, CA, USA). The ELMO3 siRNA sequence was 5'-GCGGAACGUGGUGAAGAUU-3' and the negative control was 5'-UUCUCCGAACGUGUCACGU-3'. After transfection for 24 h and 48 h, qPCR and western blot analysis was used to detect the knockdown efficiency of the ELMO3.

### 2.5. Measurement of the Cell Proliferation Rate

Cell proliferation was assayed using the Non-Radioactive Cell Proliferation Assay (3-(4,5-dimethylthiazol-2-yl)-5-(3-carboxymethoxyphenyl)-2-(4-sulfophenyl)-2H-tetrazolium,MTS) (Promega, Madison, WI, USA). Cells (5 × 10^4^/ml) were harvested after transfection and then seeded in a 96-well plate. After incubation for 1, 2, 3, 4, and 5 days, 20 *μ*l of the MTS reagent was added into each well and incubated at 37°C for 3 h. The absorbance at 490 nm was evaluated using an iMark Microplate Absorbance Reader (Bio-Rad, Hercules, CA, USA). All determinations were performed in triplicate.

### 2.6. Cell Cycle Experiment

The effect of silencing ELMO3 on the cell cycle of SGC7901 and BGC823 was assessed by flow cytometry assay. Cells were seeded in a 12-well plate at a density of 1.5 × 10^5^ cells/well overnight. We collected the cells separately after 24 h and 48 h of transfection. Then, the harvested cells were fixed in 70% ice-cold ethanol and stored at 4°C for 2 h. Next, the fixed cells were washed twice with cold PBS and then incubated with 500 *µ*l of PI (20 *µ*g/mL) (Sigma, St. Louis, MO, USA) staining solution in the dark at room temperature for 30 min. The staining solution contained 0.1% Triton X-100 (Sigma, St. Louis, MO, USA) and RNase A (Sigma, St. Louis, MO, USA)). Finally, the DNA content of the cells was measured by a BD FACSCalibur flow cytometer (Becton Dickinson, San Jose, CA, USA).

### 2.7. Wound-Healing Assay

The wound-healing assay was performed to examine cell migratory capacity. Briefly, cells were seeded and incubated in a 12-well plate until they formed a confluent monolayer. A “scratch” of the cell monolayer was scraped in a straight line with a sterile pipette tip. The cell fragments caused by the scratch were washed away with sterile PBS [28, 29]. The plate was then incubated for 24 h. After 24 h and 48 h, the cell migration into the scratch wounds was observed through a phase-contrast microscope. Each experiment was conducted in triplicate. The distances the cells moved were observed and recorded.

### 2.8. Transwell Assays

Transwell assays were used to detect whether ELMO3 knockdown affects GC cell invasion and migration and were performed as described previously, with slight modifications.

For migration assays, GC cell suspensions (0.4 ml, 1 × 10^5^) diluted in serum-free culture medium were seeded in the upper chambers (Corning Costar, Acton, MA, USA) without a coating of Matrigel. For the invasion assays, GC cells (0.4 ml, 1 × 10^5^) diluted in serum-free culture medium, were seeded on Matrigel-coated upper chambers (Corning Costar, Acton, MA, USA). In both assays, 0.6 ml RPMI Medium 1640 containing 10% FBS was added to the lower chamber [30]. After incubation for 12 h, 24 h, and 48 h, the Matrigel and the non-migrating cells in the upper chambers were carefully removed or cleaned. Cells moving to the bottom chamber were fixed with 4% paraformaldehyde for 20 min and then stained with 0.1% crystal violet solution at room temperature for 20–30 min. The cells were subsequently washed twice with PBS, and the stained cells were observed under a microscope. Three fields on each membrane were randomly selected, and the average number of penetrating cells was counted under a 100 × inverted microscope.

### 2.9. F-Actin Staining

For immunofluorescence staining, 5 × 10^4^ cells were seeded in 24-well plates containing sterile cover glasses and incubated for 24 h. Then, the cells were fixed with 4% paraformaldehyde in PBS for 10 min and permeabilized with 0.1% TritonX-100 in PBS for 5 min. After blocking in 1% BSA for 20 min, the cells were incubated with 1 mM FITC-phalloidin (Sigma, Saint Louis, MO, USA) for 40 min and then counterstained with 50 mM DAPI (Invitrogen, Life Technologies, Carlsbad, CA, USA) for 2 min in the dark at room temperature. To remove unbound phalloidin, cells were washed three times with PBS. Images were acquired with a fluorescence microscope (Nikon Eclipseni NI, Tokyo, Japan).

### 2.10. Statistical Analysis

Data collected from at least three independent experiments were expressed as the mean ± the standard deviation (SD), and representative data are shown. All statistical analyses were carried out with SPSS standard version 11.5 (SPSS Inc., Chicago, IL, USA). A one-way ANOVA was applied to evaluate the statistical differences between groups. The threshold for statistical significance was P < 0.05.

## 3. Results

### 3.1. The Abnormal Expression of ELMO3 in Human Gastric Cancer

**Figure 1:**
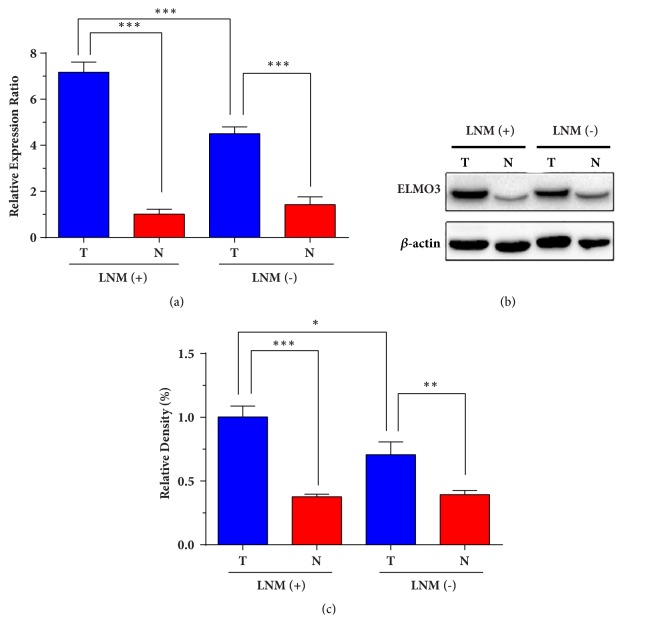
The mRNA transcript level and protein expression of ELMO3 in gastric cancer tissues and paired adjacent normal tissues. (a) The ELMO3 mRNA level was detected in primary tumors (T) and matched adjacent normal tissues (N) in GC patients by qPCR analysis. LNM (+): GC patients with lymph node metastasis; LNM (-): GC patients without lymph node metastasis. (b) The ELMO3 protein level was examined by Western blot analysis. *β*-Actin functions as an internal control to normalize ELMO3 expression. The quantitative analysis of the expression levels is presented in (c). The statistical analysis was carried out by analysis of variance (ANOVA). *∗ P* < 0.05, *∗∗ P* < 0.01, *∗∗∗ P* < 0.001.

To explore the expression pattern of ELMO3 in gastric carcinogenesis, the mRNA transcript level and protein level of ELMO3 were detected in GC tissues and paired adjacent tissues by qPCR analysis and western blot analysis. The results showed that the mRNA level of ELMO3 was significantly higher in cancer tissues than in paired adjacent tissues from 48 paired specimens (*P *= 0.003, Figure 1(a)). In line with the results of qPCR, the ELMO3 protein level in the GC cancer tissues was also significantly higher than in the paired adjacent tissues (*P *< 0.001, Figures 1(b) and 1(c)). Furthermore, for those GC patients with lymph node metastasis, the protein level of ELMO3 was markedly higher in cancer tissue than in lymph node metastasis-free patients (*P* = 0.017).

We further examined the expression of ELMO3 in five gastric cancer cell lines, including SGC7901, BGC823, MGC803, AGS, and MKN74, and in one normal gastric epithelial cell line, Ges-1. The results of qPCR and Western blot are shown in Figure S1. This revealed that ELMO3 had a higher expression level in tumor cells than in normal cells. To clarify the role of ELMO3 in gastric cancer, SGC7901 and BGC823 cell lines with higher expression of ELMO3 were used for the subsequent experiments.

In addition, RNA interference treatment was employed to inhibit the expression of ELMO3 in GC SGC7901 and BGC823 cells in the following experiments. An ELMO3-specific siRNA (siELMO3) sequence was used as previously described [23], and it resulted in approximately 75% knockdown efficiency in the two cell lines (data not shown).

### 3.2. The Aberrant Expression of ELMO3 Contributes to Cell Proliferation in GC Cells

**Figure 2:**
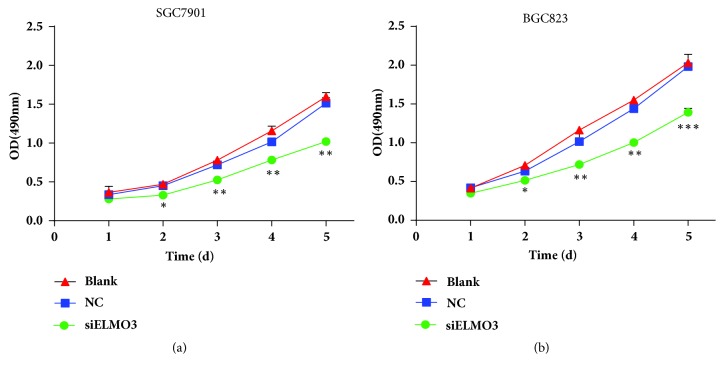
Silencing ELMO3 inhibited cell proliferation in GC cells. An MTS assay was conducted to determine the cell proliferation ability of GC SGC7901 cells (a) and BGC823 cells (b). NC represents the negative control siRNA. The transfection reagent Lipofectamine™ 2000 was used as a blank control. The values represent the mean ± SD of the absorbance at various time points (n = 6, analysis of variance of factorial design). *∗ P* < 0.05, *∗∗ P* < 0.01, *∗∗∗ P* < 0.001.

To determine the effects of ELMO3 on cell proliferation, an MTS assay was performed to explore the potential role of ELMO3 in GC cell proliferation (Figure 2). The results indicated that silencing ELMO3 markedly reduced the viability of GC SGC7901 and BGC823 cells compared with their corresponding negative and blank controls at 1–5 days. This indicates that the high expression of ELMO3 in GC increases cell proliferation.

### 3.3. Knockdown of ELMO3 Altered Cell Cycle Distribution in GC SGC7901 and BGC823 Cells

**Figure 3:**
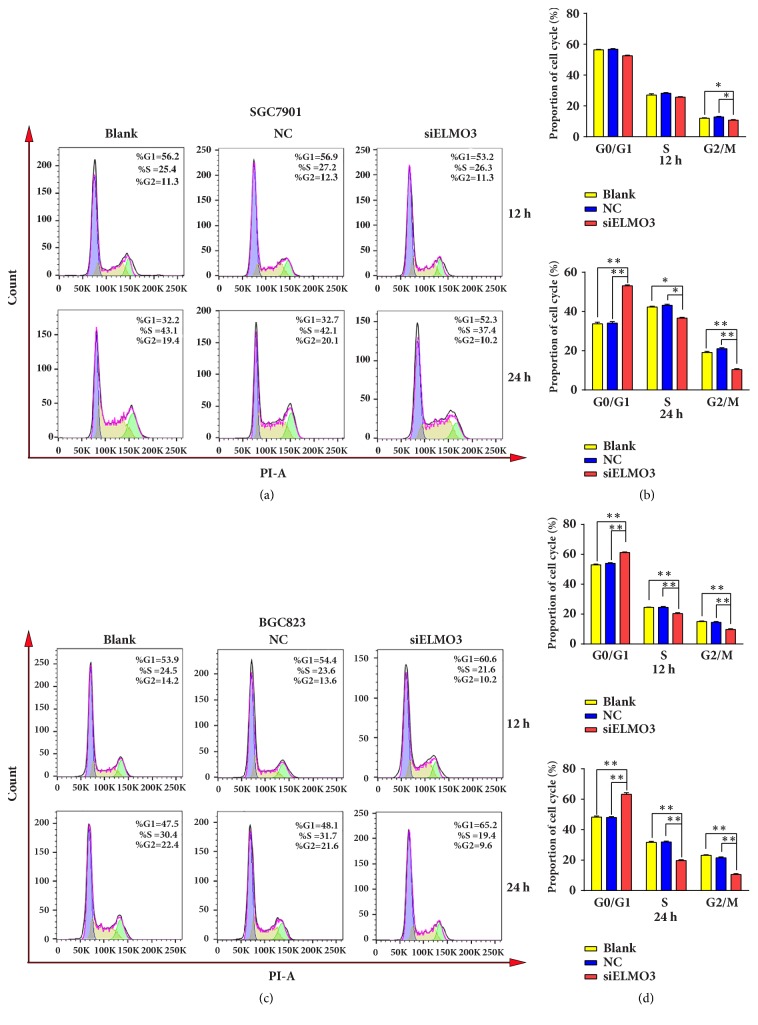
ELMO3 knockdown altered the cell cycle of GC cells. (a) The cell cycle of SGC7901 was arrested in the G1 phase. Its quantitative measurement is presented in (b). (c) The cell cycle of BGC823 was arrested in the G1 fraction. Its quantitative measurement is presented in (d). Statistical analysis was carried out by ANOVA. NC represents the negative control siRNA. *∗ P* < 0.05, *∗∗ P* < 0.01, *∗∗∗ P* < 0.001.

In cancer, genetic mutations contribute to regulatory process malfunctions of the cell cycle and result in uncontrolled cell proliferation. To evaluate whether ELMO3 is involved in the regulation of the cell cycle in GC cells, a flow cytometry experiment using propidium iodide (PI) DNA staining was performed to investigate the changes in cell cycle distribution when ELMO3 was inhibited. As shown in Figures 3(a) and 3(b), silencing ELMO3 for 24 h significantly increased the percentage of G1-phase cells compared with NC and blank control groups in SGC7901 cells, whereas siELMO3 treatment markedly decreased the percentage of S-phase and G2-phase cells compared with NC and blank control groups. Knockdown of ELMO3 led to a similar result in BGC 823 cells as in the SGC 7901 cells, which is shown in Figures 3(c) and 3(d). This suggests that the aberrant expression of ELMO3 could impact cell cycle progression in GC cells.

### 3.4. Silencing ELMO3 Suppressed Cell Migration and Invasion in GC SGC7901 and BGC823 Cells

**Figure 4:**
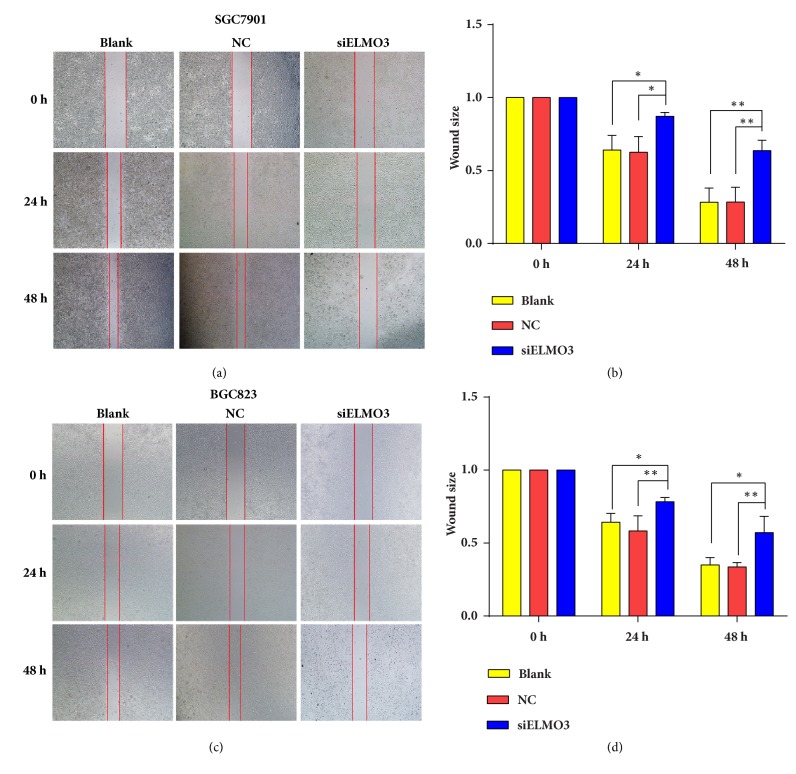
Effect of silencing ELMO3 on the cell motility rate. Scratch wound-healing assays showed that knockdown of ELMO3 suppressed cell motility in GC SGC7901 cells (a, b) and BGC823 cells (c, d) at 24 h and 48 h. The statistical analysis was performed using ANOVA. NC represents the negative control siRNA. *∗ P* < 0.05, *∗∗ P* < 0.01, *∗∗∗ P* < 0.001.

**Figure 5:**
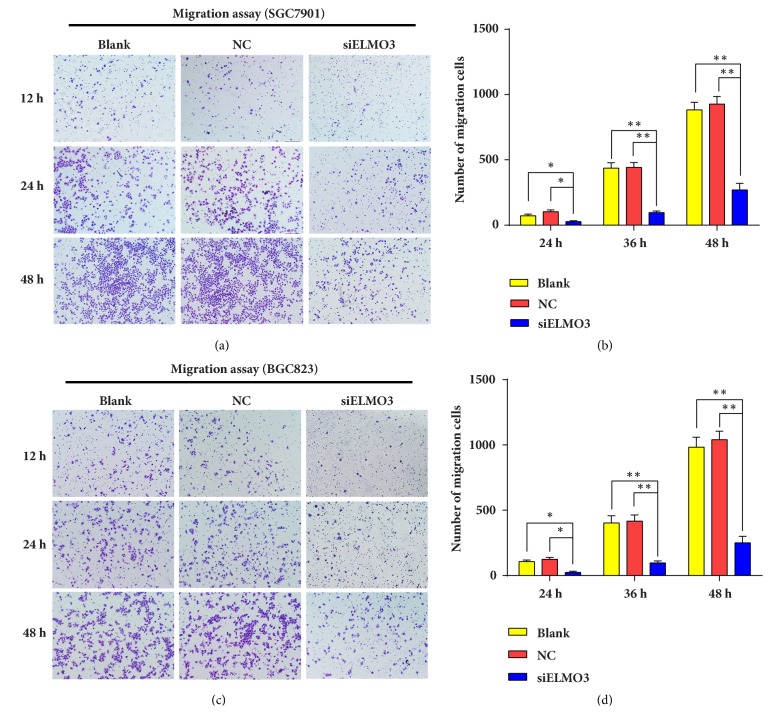
Transwell assays were developed to assess the effect of ELMO3 knockdown on the cell migratory capability of GC SGC7901 cells (a, b) and BGC823 cells (c, d). The statistical analysis was performed using ANOVA. NC represents the negative control siRNA. *∗ P* < 0.05, *∗∗ P* < 0.01, *∗∗∗ P* < 0.001.

The migration of cancer cells is a central step in cancer invasion and metastasis. To elucidate the potential role of ELMO3 in the metastasis and invasion of GC cells, wound-healing, and Transwell assays were conducted. As shown in Figure 4, the wound-healing assay results revealed that downregulating the expression of ELMO3 in GC cells significantly suppressed cell mobility at 24 h compared with that in the NC and blank groups. Similar to the results of the scratch test, knockdown of ELMO3 markedly inhibited cell migratory ability in Transwell assays. As presented in Figure 5, the number of migrated cells were markedly reduced after 12 h of siELMO3 treatment, which became evident by 24 and 48 h compared with the NC and blank groups.

**Figure 6:**
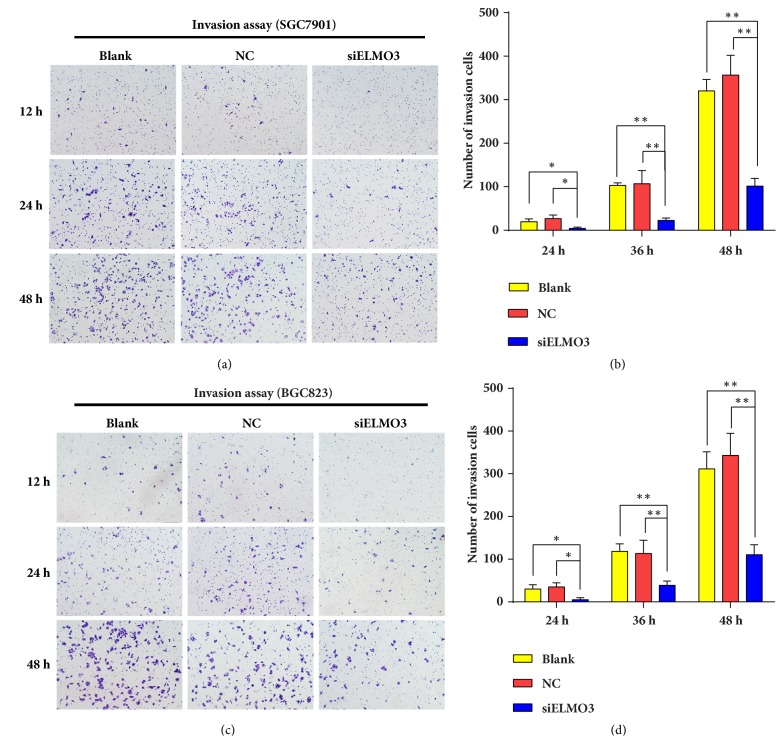
A Matrigel Transwell assay was conducted to evaluate the effect of silencing ELMO3 on the cell invasive potential of GC SGC7901 (a, b) and BGC823 cells (c, d). The statistical analysis was performed using ANOVA. NC represents the negative control siRNA. *∗ P* < 0.05, *∗∗ P* < 0.01, *∗∗∗ P* < 0.001.

Moreover, knockdown of ELMO3 also inhibited the ability of GC cells to invade through a Matrigel basement membrane. As shown in Figure 6, the number of invaded cells significantly declined with siELMO3 treatment over 12 h. Thus, silencing ELMO3 suppressed the invasion of human GC cells in vitro. Based on these results, ELMO3 has a promoting role in the regulation of GC cell migration and invasion.

### 3.5. Silencing ELMO3 Inhibits F-Actin Polymerization

**Figure 7:**
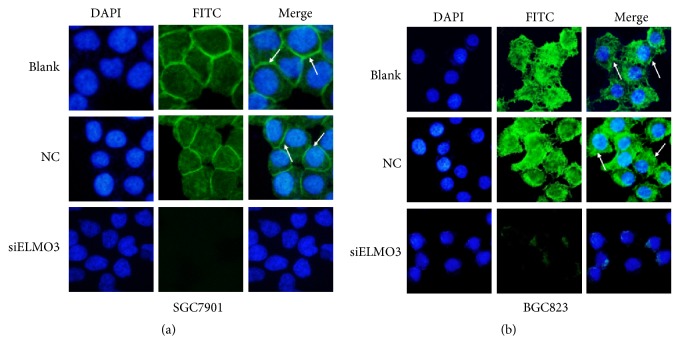
Silencing ELMO3 suppressed the F-actin polymerization of GC SGC7901 (a) and BGC823 cells (b). FITC-phalloidin and DAPI could specifically bind F-actin and nuclei. Arrows point to the cells displaying F-actin polymerization responses. The representative photographs are shown at 400× magnification. NC represents the negative control siRNA.

Cell migration is initiated by forming membrane protrusions, the driving force for which is the localized polymerization of submembrane actin filaments, whose assembly and disassembly drive cellular motility by regulating cell shape, enabling cells to protrude, polarize, and move [31]. To clarify the potential relationship between ELMO3 and actin filaments, we examined whether silencing ELMO3 could affect the cellular localization of the actin cytoskeleton in gastric cells. As illustrated in Figure 7, the knockdown group presented much fewer green fluorescent signals of F-actin structure distribution in cells compared with the NC and blank groups. This suggests that the downregulation of ELMO3 could inhibit the F-actin polymerization of SGC7901 and BGC823 cells and thus affect GC cell migration.

## 4. Discussion

Tumor invasion and metastasis are not only the essential characteristics of malignant tumors but the main reason leading to the death of most cancer patients [32]. Previous findings have indicated that the ability of cells to migrate is a key attribute required for invasion and metastasis [33]. Chemotaxis is one of the critical types of directed tumor cell migration. During this process, ELMO proteins can interact directly with the G*β* subunit of the G protein and transduce G protein-coupled receptor signals to control the reorganization of the actin-based cytoskeleton. Therefore, ELMO family proteins play an important role in regulating cell migration. ELMO1 and ELMO2 are highly expressed in most human cancer tissues, whereas ELMO3 is only found in some cancer tissues, and its function has not been fully clarified in GC.

In the present study, the expression level of ELMO3 was significantly higher in GC cancer tissues than in the matched adjacent tissues. In addition, ELMO3 was also highly expressed in tumor cell lines compared with its expression in normal gastric epithelial cells. This indicates that the high expression of ELMO could be associated with tumor development. Several previous studies report ELMO3 augmented expression parallels increased lymph node metastasis. In non-small-cell lung cancer, the ELMO3 mRNA transcript level is significantly upregulated in patients' cancer tissues and serum compared with matched adjacent tissues [19]. Further regression analysis indicated that high expression of ELMO3 can lead to a short overall survival time. Other studies such as in head and neck squamous cell carcinoma [24] and T1 laryngeal cancer [25] also reached a similar conclusion. Therefore, our results indicate that ELMO3 could play a crucial and distinct role in the development and progression of GC, which is similar to the findings of previous reports.

In the MTS assay, knockdown of ELMO3 significantly decreased cellular proliferation of GC cells, revealing that ELMO3 serves a crucial role in GC cell growth. This result is similar to a previous study of breast cancer [18], in which silencing ELMO2 suppressed tumor cell proliferation, while Axl-mediated phosphorylation of ELMO2 played a central role in promoting cell growth. Whether ELMO3 in gastric cancer cells serves a similar role as ELMO2 in breast cancer cells needs further investigation.

The results of the flow cytometry experiment showed that downregulating ELMO3 expression significantly increased the percentage of G1-phase cells and decreased the percentage of S-phase and G2-phase cells. These findings further support our hypothesis that ELMO3 is involved in the process of GC cell growth. This is also in agreement with our previous report on colorectal cancer, in which silencing ELMO3 significantly induced G1 cell cycle arrest [23]. ELMO3 inhibition could be a promising option for slowing or preventing the progression of GC. Further studies should be conducted to address the specific role of ELMO3 in the development of GC.

The results of wound-healing and Transwell assays revealed that knockdown of ELMO3 could inhibit the invasion and migration of SGC7901 and BGC823 cells, which is consistent with previous studies in colorectal cancer [23]. However, the related molecular mechanism regulating cell migration is still unclear. Substantial evidence shows that ELMO family members ELMO1 and Dock180 are coexpressed as a complex to promote cell migration through activating Rac in studies from* C. elegans* to mammals [15, 34, 35]. In breast cancer cells, Axl can phosphorylate ELMO1/2 to promote Rac activation and cell invasion [18]. Because ELMO family proteins possess similar Elmo-domains (~70 aa C-terminal domains) and are conserved throughout evolution [10], it is possible that ELMO3 is involved in a similar mechanism in modulating cell invasion and migration with ELMO1 and ELMO2. In addition, DNA methylation changes in gene promoter CpG islands are usually considered to affect the expression of tumor-related genes and influence cancer cell motility. In non-small-cell lung cancer, the high expression level of ELMO3 coincides with low methylation levels of its promoter region, contributing to the formation of metastases. In colorectal cancer Caco-2 cells, the activity of the ELMO3 promoter can be regulated by caudal-related homeobox transcription factor 2 (CDX2), which interacts with SP1 [36]. However, no significant association between increased promoter methylation levels of ELMO3 and an impact on patients' outcomes in glioblastoma has been seen [26]. Therefore, the mechanism whereby ELMO3 regulates GC cell invasion and metastasis could be very complicated and possibly differs in different tumor types.

Cytoskeletal proteins are essential for maintaining cellular morphology and cell motility in eukaryotic cells. F-actin serves as an important component of the cytoskeleton. Polymerization of F-actin alters leading edge cell shapes and promotes cell migration [32, 37]. In the present study, the downregulation of ELMO3 inhibited the F-actin polymerization of GC cells and thus affected the cellular motility. Our result is similar to those of previous studies of ELMO1 and ELMO2, in which ELMO1, ELMO2, and Dock were found to play a significant role in promoting the growth of actin polymerization to drive cell migration [38, 39]. Furthermore, ELMO proteins as essential components of the ELMO/Dock complex directly interact with G protein and transduce GPCR signaling to control dynamic remodeling of the actin cytoskeleton by regulating Rac activation during chemotaxis [5, 12]. However, how ELMO3 participates in regulating reorganization of the actin cytoskeleton remains unknown. Whether ELMO3 can form a complex with Dock180 to regulate Rac activation in tumor cell migration requires more evidence.

## 5. Conclusion

In this study, we demonstrated that ELMO3 expression was closely related to GC progression in patients. ELMO3 participated in the cell growth, invasion, and metastasis of GC, and it also influenced actin polymerization. Future studies are needed to explore the molecular mechanism by which ELMO3 regulates invasion and metastasis. ELMO3 is expected to be a new predictive biomarker for diagnosis and serves as a potential target for the treatment of gastric cancer.

## Data Availability

The data used to support the findings of this study are available from the corresponding author upon request.

## Supplementary Materials

Supplementary Materials Supplementary materials contain the figure that showed the expression level of ELMO3 in gastric cancer cell lines (Supplemental figure 1, Figure S1).
