# Supplementary material for: Silencing ELMO3 Inhibits the Growth, Invasion, and Metastasis of Gastric Cancer
Source: Biomed Res Int. 2018 Sep 24;2018:3764032. doi: 10.1155/2018/3764032 (PMC6174816; doi:10.1155/2018/3764032)
Supplement: Supplementary Materials — contain the figure that showed the expression level of ELMO3 in gastric cancer cell lines (Supplemental figure 1, Figure S1). [file 3764032.f1.pdf]

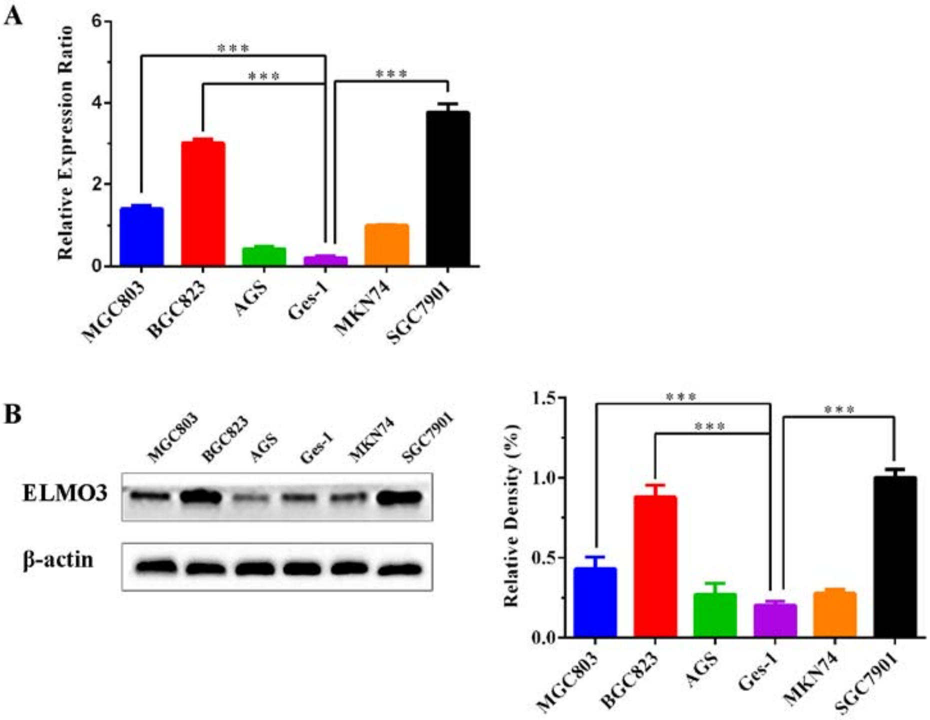

Figure S1: The expression level of ELMO3 in GC cell lines. (**A**) The mRNA level was detected by qPCR analysis. (B) The protein level was determined by Western blot analysis. β-actin served as an internal control to normalize ELMO3 expression. The statistical analysis was carried out by analysis of variance (ANOVA). *** $P < 0.001$.
